# Failure of levofloxacin treatment in community-acquired pneumococcal pneumonia

**DOI:** 10.1186/1471-2334-5-106

**Published:** 2005-11-24

**Authors:** Andrea Endimiani, Gioconda Brigante, Alessia A Bettaccini, Francesco Luzzaro, Paolo Grossi, Antonio Q Toniolo

**Affiliations:** 1 Laboratory of Microbiology and Virology, University of Insubria and Ospedale di Circolo e Fondazione Macchi, Varese, Italy; 2 Department of Infectious Diseases, University of Insubria and Ospedale di Circolo e Fondazione Macchi, Varese, Italy

## Abstract

**Background:**

*Streptococcus pneumoniae *is the leading cause of community-acquired pneumonia (CAP). High global incidence of macrolide and penicillin resistance has been reported, whereas fluoroquinolone resistance is uncommon. Current guidelines for suspected CAP in patients with co-morbidity factors and recent antibiotic therapy recommend initial empiric therapy using one fluoroquinolone or one macrolide associated to other drugs (amoxicillin, amoxicillin/clavulanate, broad-spectrum cephalosporins). Resistance to fluoroquinolones is determined by efflux mechanisms and/or mutations in the *parC *and *parE *genes coding for topoisomerase IV and/or *gyrA *and *gyrB *genes coding for DNA gyrase. No clinical cases due to fluoroquinolone-resistant *S. pneumoniae *strains have been yet reported from Italy.

**Case presentation:**

A 72-year-old patient with long history of chronic obstructive pulmonary disease and multiple fluoroquinolone treatments for recurrent lower respiratory tract infections developed fever, increased sputum production, and dyspnea. He was treated with oral levofloxacin (500 mg bid). Three days later, because of acute respiratory insufficiency, the patient was hospitalized. Levofloxacin treatment was supplemented with piperacillin/tazobactam. Microbiological tests detected a *S. pneumoniae *strain intermediate to penicillin (MIC, 1 mg/L) and resistant to macrolides (MIC >256 mg/L) and fluoroquinolones (MIC >32 mg/L). Point mutations were detected in *gyrA *(Ser81-Phe), *parE *(Ile460-Val), and *parC *gene (Ser79-Phe; Lys137-Asn). Complete clinical response followed treatment with piperacillin/tazobactam.

**Conclusion:**

This is the first Italian case of community-acquired pneumonia due to a fluoroquinolone-resistant *S. pneumoniae *isolate where treatment failure of levofloxacin was documented. Molecular analysis showed a group of mutations that have not yet been reported from Italy and has been detected only twice in Europe. Treatment with piperacillin/tazobactam appears an effective means to inhibit fluoroquinolone-resistant strains of *S. pneumoniae *causing community-acquired pneumonia in seriously ill patients.

## Background

*Streptococcus pneumoniae *is the leading cause of community-acquired pneumonia (CAP) and a major cause of meningitis and otitis media. Recent reports show an high global incidence of macrolide and penicillin resistance, whereas fluoroquinolone (FQ) resistance is not frequent [1-3]. Current guidelines for suspected bacterial CAP in patients with co-morbidity factors and recent antibiotic therapy recommend initial empiric therapy using one respiratory FQ or one macrolide associated to other drugs (amoxicillin, amoxicillin/clavulanate, broad-spectrum cephalosporins) [4,5].

Resistance to FQ in *S. pneumoniae *is determined by efflux mechanisms and/or mutations in the quinolone resistance-determining regions (QRDRs) of *parC *and *parE *genes coding for topoisomerase IV and/or *gyrA *and *gyrB *genes coding for DNA gyrase [6]. The most frequent resistance mechanism detected in *S. pneumoniae *is represented by associated mutations of both *gyrA *and *parC *genes [1,3,7]. A single *parC *mutation appears to represent the first step conferring low-level FQ resistance [6]. A second mutational step in the *gyrA *gene produces high-level resistance to FQ [6]. Both mutations are probably selected in response to the widespread use of FQ [8,9]. Ciprofloxacin and levofloxacin use is commonly associated with *parC *gene mutations, whereas use of other FQ appears to favor the selection of mutations in other genes [6]. Here, we report the first Italian case of CAP in which failure of levofloxacin treatment was associated to high-level FQ resistance in *S. pneumoniae*.

## Case presentation

### Microbiological methods

Bacterial identification (ID) and antimicrobial susceptibility testing (AST) were achieved with the Phoenix System using SMIC/ID-2 panels (Becton Dickinson Diagnostic Systems, Sparks, MD). Specie ID was confirmed using optochin and bile solubility tests (Taxo Disc, BBL, Becton Dickinson) [10]. Determinations of minimal inhibitory concentration (MIC) were obtained with the Etest method (AB Biodisk, Solna, Sweden) on Mueller-Hinton agar with 5% sheep blood (Oxoid, Milano, Italy). MIC values were interpreted according to current criteria of the Clinical and Laboratory Standards Institute [11].

Amplification of *gyrA*, *gyrB*,*parC*, *parE*, and pneumolysin genes were performed by PCR using reported oligonucleotides [12-14]. Direct DNA sequencing was obtained using the ABI Prism Big Dye terminator Cycle Sequencing Kit and the ABI Prism 310 sequencer (Applied Biosystems, Foster City, CA). DNA sequences were compared with sequences present in the GenBank database (*gyr*A, accession No. AB010387; *gyr*B, accession No. Z67740; *par*E and *par*C, accession No. Z67739). Efflux mechanisms were investigated by broth dilution in Mueller-Hinton medium containing 5% sheep blood using serial dilution of ciprofloxacin in the presence or in the absence of 10 mg/L reserpine (Sigma-Aldrich, St. Louis, MO). A fourfold or greater decrease of the ciprofloxacin MIC in the presence of reserpine was considered as indicator of efflux mechanisms [15].

### Clinical data and results

In March 2005, a 72-year-old patient with long history of chronic obstructive pulmonary disease (COPD), congenital bullous emphysema, and aortic valve incompetence developed abrupt-onset fever, cough, increased sputum production, and dyspnea. Over the past few years, solely on the basis of clinical data, the patient had received multiple treatments with oral levofloxacin and ciprofloxacin for recurrent lower respiratory tract infections (LRTi). At the onset, the family physician prescribed oral levofloxacin (500 mg, bid) and paracetamol (500 mg, tid). Three days later, because of non-remitting fever and acute respiratory insufficiency, the patient was admitted to the Emergency Room of the Ospedale di Circolo e Fondazione Macchi (Varese, Italy). On the basis of physical examination and hypoxemia (pO_2 _= 45.4 mm Hg) the patient was intubated. A central venous catheter was implanted. Blood analysis showed the following results: erythrocytes 4.23 × 10^6^/μL, hemoglobin 13.3 g/dL, hematocrit 38.8%, platelets 201 × 10^3^/μL, total leukocytes 15.75 × 10^3^/μL (neutrophils 78.2%, lymphocytes 12.1%), ESR (erythrocyte sedimentation rate) 86.2 mm/h, CRP (C-reactive protein) 118 mg/L, creatinine 1.02 mg/dL, BUN (blood urea nitrogen) 26 mg/dL. Chest radiograph showed accentuated peribronchial interstitium and hilar expansion. Intravenous corticosteroids and bronchodilators were given. Twelve hours later, the patient was transferred to the Intensive Care Unit (ICU) where 4 bronchoalveolar lavages (BAL) were obtained. Levofloxacin was continued by the i.v. route (500 mg, bid) supplemented with piperacillin/tazobactam (2.25 g tid). Serial BAL were also obtained during the first 3 days of hospitalization.

Microbiological tests detected a multidrug-resistant (MDR) *S. pneumoniae *isolate (10^6 ^colony forming units/mL) in the four BAL samples obtained at admission. Isolates had the following MIC values: penicillin G (1 mg/L), piperacillin (1 mg/L), piperacillin/tazobactam (1 mg/L), ceftriaxone (0.5 mg/L), cefotaxime (0.5 mg/L), cefepime (0.5 mg/L), imipenem (0.064 mg/L), erythromycin (>256 mg/L), claritromicyn (>256 mg/L), clindamycin (>256 mg/L mg/L), levofloxacin (>32 mg/L), ciprofloxacin (>32 mg/L), moxifloxacin (>32 mg/L), tetracycline (0.5 mg/L), chloramphenicol (16 mg/L), trimethoprim-sulfamethoxazole (4 mg/L), rifampin (0.5 mg/L) vancomycin (0.25 mg/L), teicoplanin (0.125 mg/L), linezolid (0.75 mg/L), and telithromycin (≤0.006 mg/L).

By direct DNA sequencing, point mutations in the *gyrA *(Ser81-Phe) and *parE *(Ile460-Val) genes were detected in all isolates. Two point mutations of the *parC *gene (Ser79-Phe; Lys137-Asn) were also present. No mutations were detected in the *gyrB *gene. The reserpine response assay failed to evidence FQ efflux. The pneumolysin gene was detected in all isolates.

Prompt clinical response followed the empirical treatment given at the ICU. On day-two, the patient was afebrile, hypoxemia resolved (pO_2 _= 157 mm Hg), inflammatory markers were decreased (total leukocytes 7.19 × 10^3^/μL, neutrophils 76.1%, lymphocytes 12.0%, ESR 45 mm/h, CRP 57 mg/L), and blood cultures were negative. BAL samples obtained on day-2 and at subsequent times were negative. Five days after hospital admission, levofloxacin was suspended and piperacillin/tazobactam was reduced (1.25 g tid). Respiratory assistance was stopped on day-12. Antibiotic treatment ended on day-14. Complete recovery ensued and the patient was discharged on day-16.

## Conclusion

The impact of antibiotic resistance on the treatment outcome of patients with CAP is a matter of discussion [4]. FQ are often the primary choice for empirical treatment of patients with co-morbidity factors such as COPD, diabetes, and renal or congestive heart failure [4,5]. A few reports show an association between FQ resistance and treatment failure in CAP caused by *S. pneumoniae *[16,17]. No cases have been yet reported from Italy, possibly due to the low incidence of FQ-resistant strains [3].

The present report describes the first Italian case of CAP due to a FQ-resistant strain of *S. pneumoniae*. Failure of oral levofloxacin was observed. According to current criteria [11], the isolate was MDR i.e., intermediate to penicillin, resistant to chloramphenicol, trimethoprim-sulfamethoxazole, macrolides, clindamycin and FQ. The strain showed single mutations of *gyrA *and *parE *genes associated with two point-mutations of the *parC *gene. High-level resistance to FQ resulted (MIC >32 mg/L). This justified the initial treatment failure when the patient was given levofloxacin alone. Molecular analysis showed a group of mutations that have not yet been reported from Italy [3,18]. Association of the reported mutations in *gyrA*, *parE *and *parC *genes has been detected only twice in Europe [3], but is relatively more frequent in the USA [7]. As demonstrated by studies of *S. pneumoniae *strains exposed *in vitro *to FQ, ciprofloxacin and levofloxacin use is commonly associated with *parC *gene mutations, whereas use of other FQ appears to favor the selection of mutations in other genes [6].

The reported clinical case suggests that the following precautions need to be used for patients with specific LRTi risk factors: i) before therapy, respiratory samples should be obtained to identify the responsible agents and to define antimicrobial susceptibility [16]; ii) patients previously treated with FQ may be given these drugs, but need strict clinical monitoring during the first 3 days of therapy to evaluate the clinical response [19]; iii) since only 1% of FQ-resistant *S. pneumoniae *strains are also resistant to broad-spectrum beta-lactams, seriously ill patients can be safely treated with combinations of FQ plus broad-spectrum beta-lactams [20]. Empiric treatment of CAP in patients seriously compromised using piperacillin/tazobactam (as in the reported case) appears an effective means to inhibit FQ-resistant strains of *S. pneumoniae*. In particular, piperacillin/tazobactam appears indicated for ICU patients since, in contrast to monotherapy with extended-spectrum cephalosporins (e.g., ceftriaxone, cefotaxime), does not favor the selection of ESBL-positive enterobacteria and/or chromosomal beta-lactamase hyperproducers (e.g., *Pseudomonas aeruginosa*) [21,22]. Additionally, this drug's spectrum is wider than that of 3^rd^–4^th ^generation cephalosporins.

In Italy FQ consumption is higher than in the rest of Europe [8]. Thus, it seems possible that over the next years an increased prevalence of FQ-resistant *S. pneumoniae *strains will be observed. For this reason the detection of a community-acquired MDR isolate of *S. pneumoniae *is of special concern. Emerging resistance traits in this species underline the need of in vitro tests based on MIC data in order to select the most appropriate drugs for preventing the dissemination of epidemic clones.

## Competing interests

The author(s) declare that they have no competing interests.

## Authors' contributions

AE and GB performed microbiological tests, analyzed the data and prepared the manuscript.

AAB carried out molecular assays.

PG diagnosed, cured and provided clinical data of the patient.

FL and AQT coordinated the study and helped writing the manuscript.

All authors read and approved the final version of the manuscript.

## Pre-publication history

The pre-publication history for this paper can be accessed here:


